# Atypical Membrane-Anchored Cytokine MIF in a Marine Dinoflagellate

**DOI:** 10.3390/microorganisms8091263

**Published:** 2020-08-20

**Authors:** Maëlle Jaouannet, Anne-Sophie Pavaux, Sophie Pagnotta, Olivier Pierre, Claire Michelet, Sophie Marro, Harald Keller, Rodolphe Lemée, Christine Coustau

**Affiliations:** 1 Institut Sophia Agrobiotech, Université Côte d’Azur-INRAE-CNRS, F-06903 Sophia Antipolis, France; maelle.jaouannet@inrae.fr (M.J.); olivier.pierre@inrae.fr (O.P.); ewilan06@hotmail.fr (C.M.); harald.keller@inrae.fr (H.K.); 2 Laboratoire d’Océanographie de Villefranche, Sorbonne Université, CNRS, F-06230 Villefranche-sur-Mer, France; pavaux@obs-vlfr.fr (A.-S.P.); sophie.marro@obs-vlfr.fr (S.M.); lemee@obs-vlfr.fr (R.L.); 3 Centre Commun de Microscopie Appliquée (CCMA), Université Côte d’Azur, F-06108 Nice, France; sophie.pagnotta@univ-cotedazur.fr

**Keywords:** MIF, *Lingulodinium polyedra*, transmembrane protein, dinoflagellate, stress response, secretion

## Abstract

Macrophage Migration Inhibitory Factors (MIF) are pivotal cytokines/chemokines for vertebrate immune systems. MIFs are typically soluble single-domain proteins that are conserved across plant, fungal, protist, and metazoan kingdoms, but their functions have not been determined in most phylogenetic groups. Here, we describe an atypical multidomain MIF protein. The marine dinoflagellate *Lingulodinium polyedra* produces a transmembrane protein with an extra-cytoplasmic MIF domain, which localizes to cell-wall-associated membranes and vesicular bodies. This protein is also present in the membranes of extracellular vesicles accumulating at the secretory pores of the cells. Upon exposure to biotic stress, *L. polyedra* exhibits reduced expression of the MIF gene and reduced abundance of the surface-associated protein. The presence of LpMIF in the membranes of secreted extracellular vesicles evokes the fascinating possibility that LpMIF may participate in intercellular communication and/or interactions between free-living organisms in multispecies planktonic communities.

## 1. Introduction

Macrophage Migration Inhibitory Factors (MIF) are pivotal cytokines/chemokines for vertebrate immune systems. They are involved in numerous infectious diseases and pathological disorders, such as septic shock, cancer, and rheumatic and cardiovascular diseases [1,2,3,4]. These multifunctional proteins control major cell functions such as migration, proliferation, and p53-mediated apoptosis [1,5,6]. MIFs are conserved across plant, fungal, protist, and metazoan kingdoms [7], but their functions have not been determined in most phylogenetic groups. The MIF proteins that have been described to date are small, conserved single-domain proteins (approx. 12.5 kDa). In a recent survey across 800 species of plants, protists, fungi, and metazoan, we reported the existence of typical MIF proteins in all kingdoms and phylogenetic groups [7]. Interestingly, we also identified a few atypical MIF proteins, including a putative membrane-anchored MIF protein from the Genbank TSA database (Genbank accession number JO713726.1) in the dinoflagellate *Lingulodinium polyedra,* but their existence remained uncertain as they may result from assembly or automatic annotation errors [7]. The possible occurrence of a transmembrane cytokine in a free living unicellular eukaryote would be unexpected, as MIFs are typically soluble proteins that are stored in vesicles of immune and non-immune cells, and massively secreted upon challenge [3,8,9,10,11]. In addition, previous studies on MIFs from unicellular species have been restricted to parasitic protists [12] such as *Plasmodium* species [13], *Leishmania*, [14], or *Entamoeba histolitica* [15]. MIFs from these species are secreted into the blood of the vertebrate host and participate in immune evasion and pathogenesis [12,16]. The aim of this study was therefore to investigate the existence of a transmembrane MIF protein in the dinoflagellate *L. polyedra*, and to start exploring its potential function.

## 2. Materials and Methods

### 2.1. Lingulodinium polyedra

The dinoflagellate *Lingulodinium polyedra* (MCCV 130) was originally collected from the “Baie de Vilaine” and cultured at the Mediterranean Culture Collection of Villefranche (MCCV). Cultures were maintained in 75 mL of L1 medium [17] prepared with sterile and aged sea water adjusted to a salinity of 34. Cultures were maintained at 22 °C, under a 14/10 light/dark cycle with a light intensity of 250 μmol m^−2^.s^−1^. Cells of *L. polyedra* used for the experiments were collected from the cultures after 14 days. They were counted in 12 mL aliquots (triplicates) fixed with acidic lugol (4% *v*/*v*) using a liquid particle counter (HIAC/Royco 9703, Pacific Scientific Instruments, Jakarta Barat, Indonesia) with a size range of 2–80 μm^2^.

### 2.2. Copepods

The harpacticoid copepod *Sarsamphiascus* cf. *propinquus* (Sars, 1906) was collected from the Marinières site (Bay of Villefranche-sur-Mer, N-W Mediterranean, 43°42′21.51″ N–7°19′07.44″ E) using a WP2 net towed over the macroalgal cover. Copepods were maintained in 10 L tanks in 0.2-μm filtered aged seawater (salinity 38) at 22 °C in the dark. They were fed three times a week with a mixture of the microalgae *Dunaliella salina* (MCCV 020) and *Tisochrysis lutea* (CCAP 927/14). 

### 2.3. cDNA Sequencing and Expression Studies

For RNA extraction, *L. polyedra* were flash-frozen and ground using the TissueLyser LT (Qiagen Inc., Venlo, The Netherlands) for 2 min at 50Hz. Samples were centrifuged at 13,000× *g*, for 20 min, at 4 °C to remove debris. Total RNA was extracted with the PureLink™ RNA Mini Kit (Ambion-Life Technologies/Thermo Fisher Scientific, Waltham, MA, USA) following the manufacturer’s instructions and were quantified using a Nanodrop (Life Technologies/Thermo Fisher Scientific, Waltham, MA, USA). RNAs were reverse-transcribed using iScript™ cDNA Synthesis Kit (Bio-Rad Laboratories, Hercules, CA, USA) for 30 min at 42 °C. The reverse transcriptase was inactivated for 5 min at 85 °C. The cDNAs were amplified by Phusion DNA Polymerase (New England Biolabs, Ipswich, UK) with the 5× Phusion GC Buffer (New England Biolabs, Ipswich, UK) and specific primers designed according to the GenBank sequence JO713726.1. Amplified cDNA was sequenced by Eurofins and deposited as LpMIF under the GenBank accession number MN911288.

For RT-qPCR, transcripts were amplified by Takyon™ qPCR SYBR^®^ MasterMix (Eurogentec, Seraing, Belgium, #UF-NSMT-B0701) following the manufacturer’s instructions. Primers for RT-qPCR were designed with Primer3Plus at www.bioinformatics.nl [18]. Primer sequences are shown in the Table S1. Expression levels of LpMIF were normalized to expression levels of internal reference genes encoding, *L. polyedra* actin (Lpactin, GenBank accession number AY423582.1), and glyderaldehyde-3-phosphate dehydrogenase (LpGAPDH, GenBank accession number AY028562.1). Both genes were validated as internal control for gene expression studies in other dinoflagellate species [19,20]. Amplification efficiencies were assessed for each amplicon and the relative expression ratios were calculated using the relative quantification method [21]. 

### 2.4. Sequence Analysis

The physico-chemical parameters of molecular weight and theoretical isoelectric point (pI) of the deduced protein were computed using the ProtParam tool (http://web.expasy.org/protparam/) [22]. The functional domains and important sites of the protein were predicted by InterPro [23] and PFAM [24] softwares. The transmembrane regions and their topology were predicted using a set of 5 transmembrane prediction algorithms: TMHMM Server [25], HMMTOP [26], TMpred [27], ∆G Prediction server [28] and Phobius [29].

### 2.5. Plasmid Design for Agrobacterium-Mediated Transformation-Subcellular Localization in an Heterologous System

LpMIF with and without the transmembrane domain, were amplified from *L. polyedra* cDNA with Gateway-compatible adaptor primers, cloned into the entry vector pDON207 (Invitrogen™/Thermo Fisher Scientific, Waltham, MA, USA) and transferred into the destination GFP fusion vector pK7WGF2 (Plant Systems Biology, VIB, Gent, Belgium, [30]) using the BP and LR reaction protocols (Invitrogen™/Thermo Fisher Scientific, Waltham, MA, USA, #11789020 and #11791100). All the constructs were validated by sequencing (Eurofins GATC, Konstanz, Germany) and transformed into *Agrobacterium tumefaciens* strain GV3301. Transformants were selected using gentamycin (25 µg/mL (Sigma-Aldrich, Saint Louis, MO, USA, #G1264)), rifampicin (50 µg/mL (Sigma-Aldrich, Saint Louis, MO, USA, #R7382)) and spectinomycin (100 µg/mL (Duchefa Biochemies, Haarlem, The Netherlands, #S0188)). Recombinant strains were grown in YEB medium with the above-mentioned antibiotics at 28 °C to an OD600 of 1.5. After centrifugation, the pellet was recovered in infiltration buffer (150 µM MES hydrate pH5.7 (Sigma-Aldrich, Saint Louis, MO, USA, #M2933), 100mM MgCl2, 100 µM acetosyringone (Sigma-Aldrich, Saint Louis, MO, USA, # D134406) [31].

Agrobacterium-mediated transient expression was carried in *N. benthamiana.* Epidermal leaf cells were syringe-infiltrated with *A. tumefaciens* at a final OD_600_ = 0.1, according to a classical procedure described classically such in Evangelisti et al. [32]. Two days after infiltration, leaf discs were observed by a laser confocal microscope (Carl Zeiss MicroImaging GmbH, Jena, Germany, #LSM880) using an excitation at 488 nm.

### 2.6. Western Blots

*L. polyedra* cells were transferred to 300 µL of protein extraction buffer (100 mM Tris pH7.4, 10 µM KCl, 10 µM EDTA, 0.1% Tergitol (Sigma-Aldrich, Saint-Louis, MO, USA, #NP40S) and Plant Protease Inhibitor Mix (Sigma-Aldrich, Saint Louis, MO, USA, #P9599). Samples were ground using the TissueLyser LT (Qiagen Inc., Venlo, The Netherlands) during 2 min at 50Hz. After 20 min under gentle rotation on ice, tubes were centrifuged at 13,000× *g*, during 20 min, at 4 °C, to remove cell debris. The supernatant was transferred to an ultracentrifugation tube and centrifuged at 100,000× *g* in a fixed angle rotor, during 1 h at 4 °C to pellet. The pellets, containing cell membranes, were washed with 1mL of extraction buffer and resuspended in 100 µL of 10% SDS and 250 µL of extraction buffer. For the immunodetection of proteins excreted in the sea water, 5000 cells of *L. polyedra* were separated from sea water after a centrifugation (4,500× *g*, 2 min). 50 mL sea water, were desalted and concentrated by successive centrifugations on Centricon tubes (Vivaspin-6, 5000 MWCO, Sartorius, Göttingen, Germany, #VS0611), to a final volume of 200 µL. Protein concentrations were measured using the Pierce™ 660nm Protein Assay (#22662) according to manufacturer’s instructions. Ten micrograms of proteins were loaded on 12% Mini-PROTEAN^®^ TGX™ precast gels (Bio-Rad Laboratories, Hercules, CA, USA, #4561045) using Laemmli buffer 2× without reducing agent (Bio-Rad Laboratories, Hercules, CA, USA, #1610737), and separated under non-denaturing conditions, to allow potential visualization of MIF oligomers. Proteins were then transferred to a 2 µm PVDF membrane (Bio-Rad Laboratories, Hercules, CA, USA, #1704157EDU) using a semi-dry blotting system. Blots were blocked 1h at room temperature (RT) with 5% milk/ PBS-T and then incubated in 5% milk/ PBS-T, over-night at 4 °C with a custom-made polyclonal antibody raised against two LpMIF peptides at 1:5000 or 1:1000. The design and synthesis of antigenic peptides as well as polyclonal antibody production and purification were performed by Proteogenix (http://www.proteogenix.fr/). After 3 washes with PBS-T, blots were incubated in 5% milk/PBS-T, 45 min at RT with a secondary antibody at 1:10,000 (goat anti-rabbit IgG-HRP, #AS09 602 Agrisera, Vännäs, Sweeden). Proteins were visualized using a chemiluminescence detection kit, Luminata™ Forte Western HRP substrate (Merck Millipore, Watford, UK, #WBLUF0100) following the manufacturer’s instructions.

### 2.7. Mass Spectrometry

Mass spectrometry was performed by the Protein Science Facility (SFR Biosciences, Lyon, France). Briefly, following protein precipitation step (using trichlororoacetic acid 20% in volume, overnight at 4 °C), samples were washed twice in acetone and solubilized in 8M urea. Samples were reduced (Tris(2-CarboxyEthyl)Phosphine (Sigma-Aldrich, Saint Louis, MO, USA, #C4706), 5 mM, 57 °C, 1 h), alkylated (iodoacetamide (Sigma-Aldrich, Saint Louis, MO, USA, #I1149) 10 mM, RT, 45 min), and digested overnight at 37 °C with trypsin (1/100 ratio). Peptides digest was next desalted using C18 spin column (Thermo Fisher Scientific, Waltham, MA, USA). Peptides were dried in a speed-vac and suspended in 50 µL 0.1% HCOOH before nanoLC-MS/MS analysis. Samples were analyzed using an Ultimate 3000 nano-RSLC (Thermo Fisher Scientific, Waltham, MA, USA) coupled on line with a Q Exactive HF mass spectrometer via a nano-electrospray ionization source (Thermo Fisher Scientific, Waltham, MA, USA). 1 µL of peptide mixtures was loaded on a C18 PepMap100 trap-column (300 µm ID × 5 mm, 5 µm, 100Å, Thermo Fisher Scientific, Waltham, MA, USA) for 3.0 min at 20 µL/min with 2% ACN, 0.05% TFA in H_2_O and then separated on a C18 Acclaim PepMap100 nano-column, 50 cm × 75 µm i.d, 2 µm, 100 Å (Thermo Fisher Scientific, Waltham, MA, USA) with a 60 min linear gradient from 3.2% to 40% buffer B (A: 0.1% FA in H_2_O, B: 0.1% FA in ACN) and then from 40 to 90% of B in 2 min, hold for 10 min and returned to the initial conditions in 1 min for 15 min. The total duration was set to 90 min at a flow rate of 300 nL/min. The oven temperature was kept constant at 40 °C. Sample were analysed with TOP20 HCD method: MS data were acquired in a data dependent strategy selecting the fragmentation events based on the 20 most abundant precursor ions in the survey scan (350–1600 Th). The resolution of the survey scan was 60,000 at m/z 200 Th. The Ion Target Value for the survey scans in the Orbitrap and the MS^2^ mode were set to 3E6 and 1E5 respectively and the maximum injection time was set to 60 ms for both scan modes. Parameters for acquiring HCD MS/MS spectra were as follows; collision energy = 27 and an isolation width of 2 m/z. The precursors with unknown charge state or a charge state of 1 were excluded. Peptides selected for MS/MS acquisition were then placed on an exclusion list for 20 s using the dynamic exclusion mode to limit duplicate spectra.

Proteins were identified by database searching using Sequest HT with Proteome Discoverer 2.2 software (Thermo Fisher Scientific, Waltham, MA, USA) against the uniprot *Lingulodinium polyedra* database (50 entries, June 2019) and LpMIF sequence. Precursor mass tolerance was set at 10 ppm and fragment mass tolerance was set at 0.02 Da, and up to 2 missed cleavages were allowed. Oxidation (M), acetylation (Protein N-terminus) were set as variable modification, and Carbamidomethylation (C) as fixed modification. Proteins were filtered with a fixed value PSM validator. 

### 2.8. Stress Bio-Assays

To investigate *L. polyedra* stress responses, three experimental samples were compared: (i) *L. polyedra* cells; (ii) *L. polyedra* exposed to the copepods for 24 h; and (iii) *L. polyedra* exposed to the copepods for 24 h and then maintained under normal conditions (without copepods) for a week. Each treatment was replicated 3 times. All samples were maintained in 75 mL flasks containing 50 mL of autoclaved, aged and 0.2-μm filtered seawater (salinity 34), at 22 °C. Prior to the experiments, copepods (adults and late copepodites) were collected from the culture tanks, rinsed twice in seawater and transferred to each flask (50 individuals per flask). They were left for 2 days without food to allow gut clearance and salinity acclimation before the experiments. *Lingulodinium polyedra* cells were added in each flask at a final concentration of 100 cells mL^−1^. All samples were maintained in 75 mL flasks containing 50 mL of autoclaved, aged and 0.2-μm filtered seawater (salinity 3.4%), at 22 °C. After 24 h of exposure, 2 mL of *L. polyedra* were sampled for each replicate to study the photosystem II (PSII) function using a Multi-Color Pulse-Amplitude-Modulated analyser (MC-PAM, Heinz Walz Gmbh, Effeltrich, Germany) by measuring the maximum quantum yield of PSII (Fv/Fm ratio) [33,34]. Prior to fluorescence measurement, samples were dark-acclimated at 24 °C for 15 min to re-open PSII reaction centers and relax non-photochemical quenching.

### 2.9. Immunolocalization

*L. polyedra* cells were centrifuged to remove sea water at 1000× *g* for 5min. They were fixed in a 4% formaldehyde/PBS solution (Sigma-Aldrich, Saint Louis, MO, USA, #252549) at RT for 45 min. Following fixation, cells were washed twice in PBS (Corning-Mediatech, Inc., Manassas, USA #21-040-CM) with 0.1% Triton and permeabilized during 10min with 0.25% Triton/PBS (Sigma-Aldrich, Saint Louis, MO, USA, #T9284). After 3 washes with 0.1% Triton/PBS (5 min, 1mL each), the samples were blocked with 1% BSA (Sigma Aldrich, Saint Louis, Missouri, USA, #A2153)/PBS at least 1h at RT, with gentle rotation. They were incubated with MIF antibody (1:1000) over night at 4 °C in1% BSA/PBS under agitation. *L. polyedra* were washed three times in PBS and incubated with 1:1000 goat anti-rabbit secondary antibody conjugated to Alexa-Fluor 488 (Invitrogen-Life Technologies/Thermo Fisher Scientific, Waltham, MA, USA, #A11008). After washing three times with PBS, the samples were mounted in chambered cover glass Lab-Tek II (Life Technologies/Thermo Fisher Scientific, Waltham, MA, USA, #A155411) and observed by confocal microscopy (Carl Zeiss MicroImaging GmbH, Jena, Germany, #LSM880). To control for potential non-specific labelling, two additional samples were systematically prepared: samples where the primary antibody was either omitted, or replaced by pre-immune serum. 

Images of fluorescent cells were converted by the LSM Image Browser software, ZEN 2011, Blue edition (Carl Zeiss, Jena, Germany) into JPEG files (8 bit black and white quality). Fluorescence was measured with ImageJ (cut off >10 pixels) [35]. Results are shown as the ratio of fluorescent vs total area. Nine images were analysed per condition, corresponding to the examination of approximately 20 independent dinoflagellates. All images, including those converted for ImageJ analyses, have been deposited in Mendeley Data (http://dx.doi:10.17632/5j4k6r79ys.1). 

### 2.10. Immunogold Labelling and Transmission Electron Microscopy (TEM)

For ultrastructural immunocytochemistry, cells were fixed with 4% paraformaldehyde (PAF) or 4% PAF and 0.2% glutaraldehyde (PG) in phosphate buffer 0.1 M (pH 7.4) (Sigma Aldrich, Saint Louis, MO, USA). They were then dehydrated in ethanol series and embedded in acrylic resin (LR-WHITE (Electron Microscopy Sciences- EMS, Hatfield, PA, USA) before sectioning. For immunogold labelling, grids were deposited face down on the top of small drops of the following solutions: PBS containing 50 mM NH4Cl for 10 min, PBS containing 1% BSA and twin 20 for 10 min, PBS containing the relevant mAbs in 1% BSA and tween 20 for 1 h, PBS containing 0.1% BSA for 10 min, PBS 10 min, PBS containing 1% BSA and tween 20 with 15 nm protein-A gold conjugated (PAG-15 nm Cell Microscopy Core, Utrecht, Netherland) for 30 min, PBS containing 0.1% BSA for 5 min, PBS for 5 min twice, PBS containing 1% glutaraldehyde for 5 min and distilled water for 5 min. For controls, only the primary antibody was omitted. The sections were then contrasted with uranyl acetate (4% in water) and visualized using a JEM 1400 Electron Microscope (JEOL, Tokyo, Japan) operating at 100 kV equipped with a MORADA SIS camera (Olympus, Tokyo, Japan).

### 2.11. Scanning Electron Microscopy (SEM) 

Cell pellets were fixed with a 1.6% glutaraldehyde solution in 1:1 mixture of 0.2 M sodium cacodylate buffer (pH 7.4) and artificial sea water at room temperature and then stored at 4 °C. After three washes in distilled water, cells were filtered on a 0.2 μm isopore filter (Merck Millipore, Carrigtwohill, Ireland). Samples on filters were subsequently dehydrated in a series of ethanol baths (70%, 96%, 100% three times, 15 min each). After a final bath in hexamethyldisilazane (Carl Roth GmbH, Karlruhe, Germany) (HMDS, 5 min), samples were left to dry overnight. Samples on filters were mounted on SEM stubs with silver paint and coated with platinum (3 nm) prior to observation. SEM observations were performed with a JSM-6700F SEM (JEOL, Tokyo, Japan) at an accelerating voltage of 3 kV.

### 2.12. Quantification and Statistical Analysis

All experiments were repeated three times independently. Kruskal-Wallis tests were used to compare the photosystem II activity between the different experimental conditions (control, stress, post-stress). Fluorescence signal intensities between the three different conditions (control, stress, unstressed) were compared using the Kruskal-Wallis statistical test (* = *p* ≤ 0.05, ** = *p* ≤ 0.01, *** = *p* ≤ 0.001). Data are presented as bars of relative fluorescence. Differences in gene expression levels were tested for statistical significance by one-way ANOVA and Tukey-Kramer tests (Software Prism v.5.0, GraphPad Software, San Diego, CA, USA). Expression levels of the target genes are presented as a relative expression normalized to internal reference genes and to expression in the control sample.

## 3. Results and Discussion

### 3.1. Lingulodinium polyedra Expresses a Transmembrane MIF Protein 

**Figure 1:**
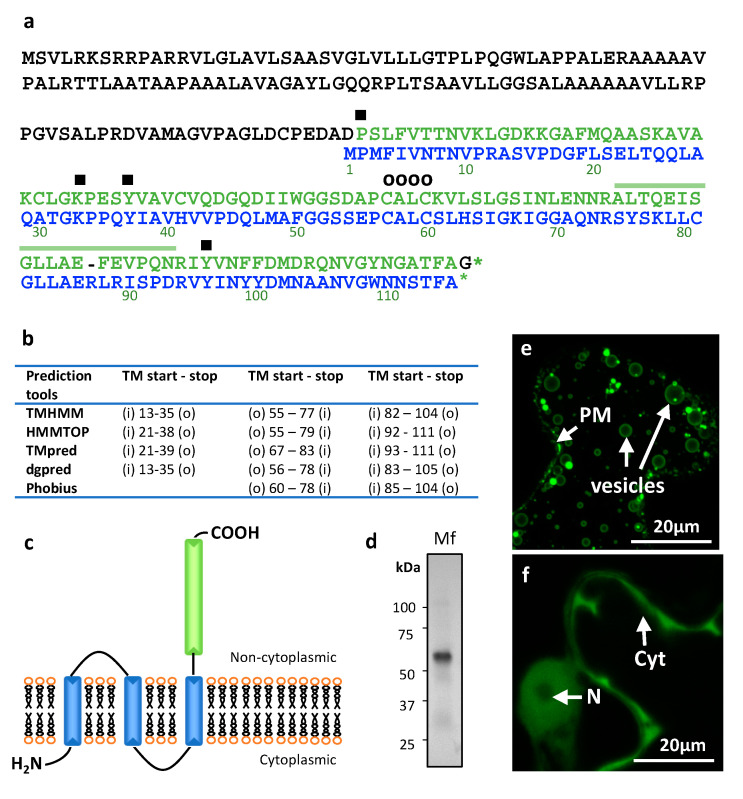
*Lingulodinium polyedra* expresses transmembrane Migration Inhibitory Factors (MIF). (**a**) Alignment of the *L. polyedra* MIF (LpMIF) sequence with human MIF (dark green). Amino acid numbers of the human MIF are given below the sequence. Hyphens (-) indicate a gap in the respective sequence. Amino acids shown to determine the tautomerase activity (■) and oxidoreductase activity (CXXC motif; O) in human MIF are shown by symbols above the sequence. The peptide identified by mass spectrometry is indicated by a line above the LpMIF sequence. (**b**) Location (LpMIF amino-acid number) and orientation of the transmembrane helices predicted by five prediction tools, where « i » refers to inside (cytoplasmic) and « o » to « outside » (non-cytoplasmic). (**c**) Schematic representation of the predicted LpMIF topology. The transmembrane domains are represented in blue and the MIF domain in green. The membrane is represented as a lipidic bilayer. (**d**) Western blot analysis of the membrane fraction (Mf) of *L. polyedra* proteins. The membrane extract (10 µg of total proteins) reveals a major protein with an apparent molecular mass of about 60 kDa. The presence of LpMIF in the most prominent band of the membrane fraction was confirmed by mass spectrometry (see 1a). (**e**,**f**) Subcellular localization of LpMIF fused to GFP in the N-terminal region, with (**e**) or without (**f**) the transmembrane domains in *Nicotiana benthamiana*. Leaves were inoculated with A. tumefaciens harboring the LpMIF-GFP constructs, 2 days prior to observations. Complete LpMIF proteins localize to the membranes of vesicles and the plasma membrane (PM) (**e**), while LpMIF without the transmembrane domains (in **f**) shows a nucleo-cytoplasmic expression in *N. benthamiana* cells, which is typical for small, soluble proteins. (N) nuclei, (Cyt) cytoplasm.

The cDNA of *L. polyedra* MIF (LpMIF) was re-sequenced and validated (Genbank accession number MN911288), thereby confirming the existence and expression of this transcript. The open reading frame encodes a 246 amino-acid protein (Figure 1a) with a predicted molecular mass of 25.07 kDa and a theoretical pI of 8.88. An MIF domain corresponding to the Interpro domain IPR001398 and PFAM domain PF01187 is predicted in the C-terminal part of the protein (positions 135 to 245) with e-values of 5.3^e−33^ and 3.2^e−27^, respectively. This domain shows 60% amino-acid similarity with the human MIF protein (Genbank accession number NP_002406.1) and harbours amino acids that have been shown to determine specific functions or activities in human MIF. Importantly, the MIF tautomerase active sites, encompassing residues Pro-2, Lys-33, Tyr-37, Ile-65, and Tyr-96, (symbol ■ in Figure 1a) are conserved, suggesting potential tautomerase activity of the LpMIF protein. Similarly, LpMIF harbours the CXXC motif (Cys-57 and Cys-60) (o symbol in Figure 1a), previously shown to determine the human MIF oxidoreductase (TPOR; thiol-protein oxidoreductase) activity [36]. This site is associated with the regulation of cellular redox homeostasis. In humans, the biological functions of these two enzymatic activities remain largely unknown, in particular because their endogenous substrates have not been identified yet [3]. However, there is increasing evidence for their roles in the pro-inflammatory effects of MIF [37]. Mutations in or inhibition of the enzymatic active sites lead to the loss of major MIF biological functions [37], possibly due to conformational changes in the catalytic domains [38]. Conservation of these active sites in LpMIF indicates that the protein could have typical MIF biological activities.

In addition to the MIF domain, transmembrane (TM) helix and general hydrophobicity predictions support the existence of three TM helices in the N-terminal part of the protein (Figure 1b). Although the exact positions of the transmembrane regions are determined slightly differently (Figure 1b), all prediction tools agree on the calculated topology, which predicts a non-cytoplasmic localization for the MIF domain (Figure 1c). As a first approach to ascertain the transmembrane nature of LpMIF, we prepared the membranes of *L. polyedra* cells and observed a major protein band (Figure 1d). The presence of an LpMIF peptide in this fraction was confirmed by mass spectrometry (Figure 1a). The apparent molecular mass for LpMIF was revealed to be approximately 60 kDa in the immunoblots, which might correspond to a dimer (expected at 50 kDa) as estimated molecular masses of proteins are not accurate under non-denaturing conditions. Typical MIF proteins can co-exist in several oligomeric forms (mono-, di-, or trimers), although MIF trimers represent the most prominent and stable form [10,39,40]. Future structural studies should reveal whether homodimers are the predominant form of the transmembrane LpMIF. Further evidence for the membrane association of LpMIF was provided by *Agrobacterium tumefaciens*-mediated transient expression assays to produce LpMIF in cells of *Nicotiana bentamiana* leaves. The use of this heterologous plant expression system confirmed that LpMIF has a non-cytoplasmic localization and accumulates in the membranes of vesicles and in the plasma membrane of the plant cells (Figure 1e). In contrast, LpMIF without the transmembrane domains shows a nucleo-cytoplasmic expression in *N. benthamiana* cells, which is typical for small, soluble proteins (Figure 1f).

### 3.2. LpMIF Is Present in the Membranes of Vesicular Bodies, Cell Wall and Extracellular Vesicles

**Figure 2:**
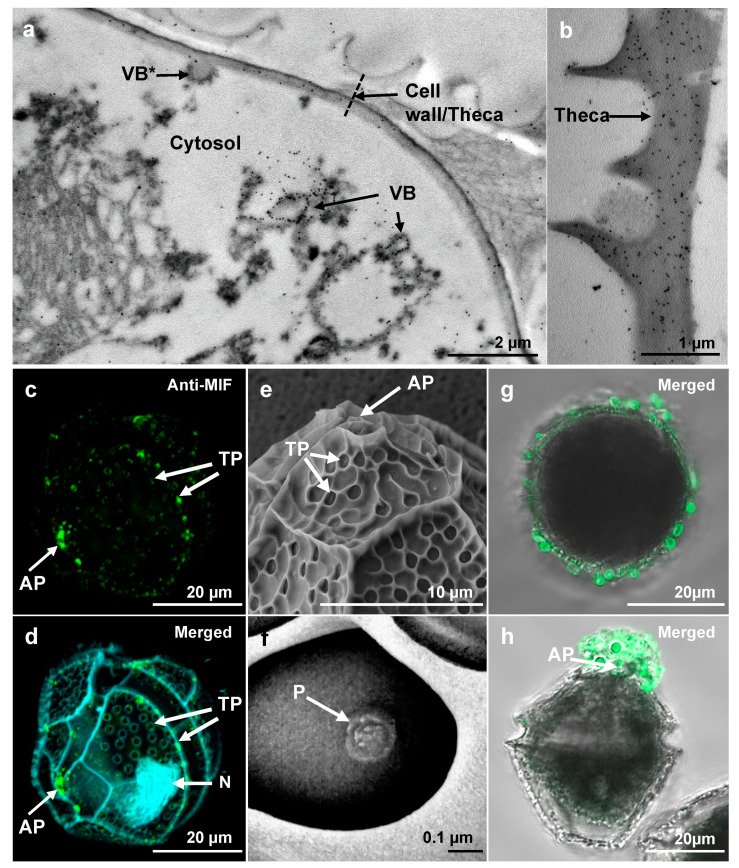
Subcellular localization of LpMIF. (**a**,**b**) TEM of immunogold-labeled *L. polyedra* cells showing the accumulation of gold particles (black dots) in membranes of the vesicular bodies (VB) and in the theca. Note that one of the vesicular bodies (indicated by a star) appears to be in a process of fusion with the cell wall. (**c**,**d**) Immunolocalization of LpMIF by confocal microscopy. LpMIF is labelled in green in (**c**,**d**), while the nucleus (N) appears in blue in (**d**) (DAPI staining). The overall structure of *L. polyedra* is visible under UV light due to autofluorescence in (**d**), and shows the shape of the thecal plates, the apical pore (AP), and the thecal pores (TP). Note the accumulation of LpMIF at the apical pore (AP) and the thecal pores (TP). (**e**,**f**) Scanning electron micrographs of *L. polyedra* showing the AP and TP. An enlargement of a TP in (**f**) shows the inner pore (P). (**g**,**h**) Representative pictures of *L. polyedra* cells that have been immunofluorescence-labelled under mild washing and fixing conditions, revealing the accumulation of MIF-labelled extracellular vesicles. Here, an apical view of the cell shows labelled vesicles surrounding the cell (**g**) and a lateral view illustrate the commonly observed accumulation of labelled mucus at the apical pore (AP). Control samples exposed to pre-immune serum or no primary antibody did not show any fluorescent signal (Figure S1e,f). Please see Supplementary Materials for procedure description.

The subcellular localization of LpMIF in *L. polyedra* cells was investigated by transmission electron microscopy (TEM) with immunogold labelling. LpMIF-bound gold particles were observed in the membrane of vesicular bodies (Figure 2a), further supporting the membrane localization of this atypical MIF protein. Extensive observation of *L. polyedra* cells did not reveal the presence of labelled LpMIF in any particular organelle (Figure 2a and Figure S1b) other than the vesicular bodies. The highest concentrations of LpMIF-bound gold particles were observed in the cell wall or theca (Figure 2b), particularly in the vicinity of the apical pore (Figure S1a). The cell wall of thecate dinoflagellates (or theca) consists of multiple membrane layers strengthened by polysaccharides [41]. This membranous composition therefore supports the presence of membrane-anchored proteins such as LpMIF. Confocal laser-scanning microscopy (CLSM) confirmed the presence of LpMIF in the theca, and further showed specific accumulation at the apical pore and at secondary thecal pores (Figure 2c,d; compare with scanning electron micrographs in Figure 2e,f). Dinoflagellates are extensively studied for their toxicity because of their roles in marine red tides and shellfish poisoning [42,43], yet their ultrastructure and physiology remain poorly understood. However, dinoflagellate pores, and apical pores in particular, are known to be involved in mucilage excretion on the cell surface [44]. In addition, thecal pores extrude fibrous and mucous trichocysts (cf Figure S1b) over the entire cell surface, which contribute to dynamic streamlining and defense against grazing [45].

To determine if LpMIF is secreted, we repeated the immunofluorescence labelling using milder washing conditions (identical procedure but without Triton). Confocal analyses subsequently showed MIF-labelled extracellular vesicles at the surface of *L. polyedra* cells (Figure 2g) with particular accumulations in mucus outside the apical pore (Figure 2h).

MIF proteins of metazoa are soluble and present in the cytoplasm of numerous cell types, stored in vesicular bodies and released in extracellular fluids (blood, haemolymph) in response to various stimuli such as bacterial lipopolysaccharides (LPS), tumour necrosis factor, or hypoxia [8,11]. Despite the decades of functional studies on human MIF, the exact mechanism of MIF secretion remains unclear. Human MIF, which does not localize to the endoplasmic reticulum or Golgi apparatus, is secreted through unconventional secretory pathways [11]. Some studies showed that MIF secretion was sensitive to inhibitors of ATP-binding cassette (ABC) transporters, while others reported the release of MIF via exosomes [3]. While the trafficking processes of LpMIF clearly requires dedicated studies, the presence of this atypical transmembrane MIF in intra- and extracellular vesicles is reminiscent of the localization of vertebrate MIFs.

### 3.3. LpMIF Is Under-Expressed during a Stress Response

**Figure 3:**
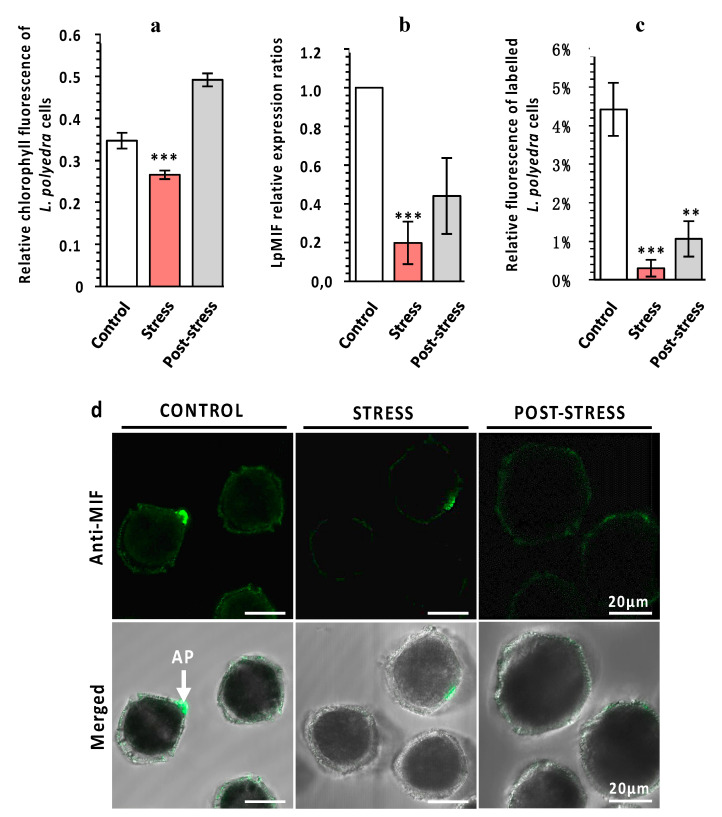
LpMIF expression during a stress response. (**a**) The exposure of *L. polyedra* to copepods decreases photosynthetic activity as a stress response. Chlorophyll fluorescence parameters were measured with a Multi-Color Pulse-Amplitude-Modulated fluorometer (Heinz Walz Gmbh, Effeltrich, Germany). Photosystem II activity was calculated from the ratio of variable fluorescence to maximum chlorophyll fluorescence (Fv/Fm). Values are means (+/− SEM) from three independent measurements/experiments. Asterisks indicate statistical differences (*** *p* < 0.001), according to Kruskal-Wallis test. (**b**) Relative expression ratios (normalized to control) of LpMIF in *L. polyedra* cells before (control), during (stress), and after (post-stress) exposure to copepods. Asterisks indicate statistical differences (*** *p* < 0.001), according to Kruskal-Wallis test. (**c**) Relative fluorescence of MIF-labelled *L. polyedra* cells expressed as the fluorescent area/total area, of cells before (control), during (stress), and after (post-stress) exposure to copepods (*n* = 9 per sample; Kruskal-Wallis test: ** = *p* ≤ 0.01, *** = *p* ≤ 0.001). (**d**) Representative pictures of MIF-labelled *L. polyedra* before (control), during (stress), and after (post-stress) exposure to copepods. Images in the upper row are fluorescence micrographs (Anti-MIF) that were merged in the lower row with transmission light micrographs (Merged). Negative control samples directly exposed to the secondary antibody are shown in the Figure S2.

Predicting putative functions for a transmembrane MIF in a free-living unicellular organism is particularly challenging. Previous studies on MIFs from unicellular species have been restricted to parasitic protists [12] such as *Plasmodium* species [13], *Leishmania* [14], *Giardia lamblia* [46], or *Entamoeba histolitica* [15]. MIFs from these species are secreted into the blood of the vertebrate host and participate in immune evasion, host invasion, and pathogenesis [12,16]. Such functions are not relevant for a free-living marine dinoflagellate. Furthermore, the major function of MIFs as innate immune regulators, which has been reported in free-living metazoan species, including invertebrates [10,16,47,48], is hardly applicable and measurable on non-model protists such as *L. polyedra*. We therefore hypothesized that LpMIF may be involved in a stress response, since functional links between immune and stress responses are well established in both animal and plant model species [49,50,51,52,53] and since MIFs from vertebrates are also involved in stress responses [2]. Under natural conditions, *L. polyedra* lives in phytoplankton communities that are exposed to grazing copepod predators. Copepods are known to induce defense responses, such as an increase in bioluminescence [54,55], a change of swimming behaviour [56], an increase of toxin production [57], and a decrease in photosynthetic activity [34,58]. It has been suggested that these induced defenses may provide slow-growing *L. polyedra* with the required advantage to co-exist with faster-growing competitors in phytoplankton communities [55]. Exposure of *L. polyedra* to the copepod *Sarsamphiascus* cf. *propinquus* for 24h correlated with a significant decrease in photosynthetic activity (Figure 3a), confirming that the copepod did induce defense responses in our experiment. This stress response is transient, since the fluorescence recovered and increased above control levels seven days after copepod exposure (Figure 3a). A decrease in LpMIF transcript content (Figure 3b) and labelled LpMIF protein content (Figure 3c,d) occurred during this stress response. This response indicates that, in this context, the transmembrane MIF from *L. polyedra* is indeed a stress-responsive protein.

## 4. Conclusions

MIFs are enigmatic proteins with extremely complex trafficking processes, biological activities, functions, and signalling pathways [3,11]. Here, we provide evidence for a new dimension in the complexity of MIF structure and possible functions. The presence of LpMIF in the membranes of secreted extracellular vesicles evokes the fascinating possibility that LpMIF participates in intercellular communication and/or regulation in the marine environment. There is increasing evidence that phytoplankton communities, including a variety of protist and bacterial species, produce substantial amounts of extracellular vesicles that are involved in interspecies communication, nutrient acquisition and exchange, biofilm formation, and cellular defense processes [59,60,61]. The multiple functions of typical MIF proteins, such as the effects on cell migration, cell division, apoptosis, and induction, of other immune or hormonal regulators, may all have a role in coordinating stress responses within a dinoflagellate colony. In addition, since MIF proteins are prone to interspecies and inter-kingdom interactions, as shown by their involvement in host–parasite interactions [16,62,63], they may also help regulate the interactions of multispecies planktonic communities. Such possibilities can now be explored by investigating LpMIF functions under biotic stress conditions.

## Supplementary Materials

The following are available online at https://www.mdpi.com/2076-2607/8/9/1263/s1, Figure S1: Immunogold-labelled electron micrographs of *L. polyedra*, Figure S2: Negative control samples of unstressed and stressed *L. polyedra*, Table S1: Accessions, names and primer sequences for transcripts analyzed in this study.
