# Supplementary material for: Atypical Membrane-Anchored Cytokine MIF in a Marine Dinoflagellate
Source: Microorganisms. 2020 Aug 20;8(9):1263. doi: 10.3390/microorganisms8091263 (PMC7565538; doi:10.3390/microorganisms8091263)
Supplement: Supplementary file 1 [file microorganisms-08-01263-s001.pdf]

Table S1. Accessions, names and primer sequences for transcripts analyzed in this study.

GenBank ID	Name	Primer Sequence (5' to 3')	Use
JO713726.1	LpMIF	ATGTCCGTGCTGCGCAAGA	full length cDNA sequencing
		TCATCCAGCGAAGGTGGCG	
JO713726.1	LpMIF	CAGGACATCATCTGGGGTGG	RT-qPCR
		GTAAATCCGGTTCTGGGGCA	
AY423582.1	Lp actin	CGAGCTGTTTTCCCCTCCAT	RT-qPCR
		TTCCATGTCGTCCCAGTTCG	
AF028562.1	Lp GAPDH	ATGGGGATCAATGGCTTCGG	RT-qPCR
		GCCATCAACTTCCTTCGTGC	

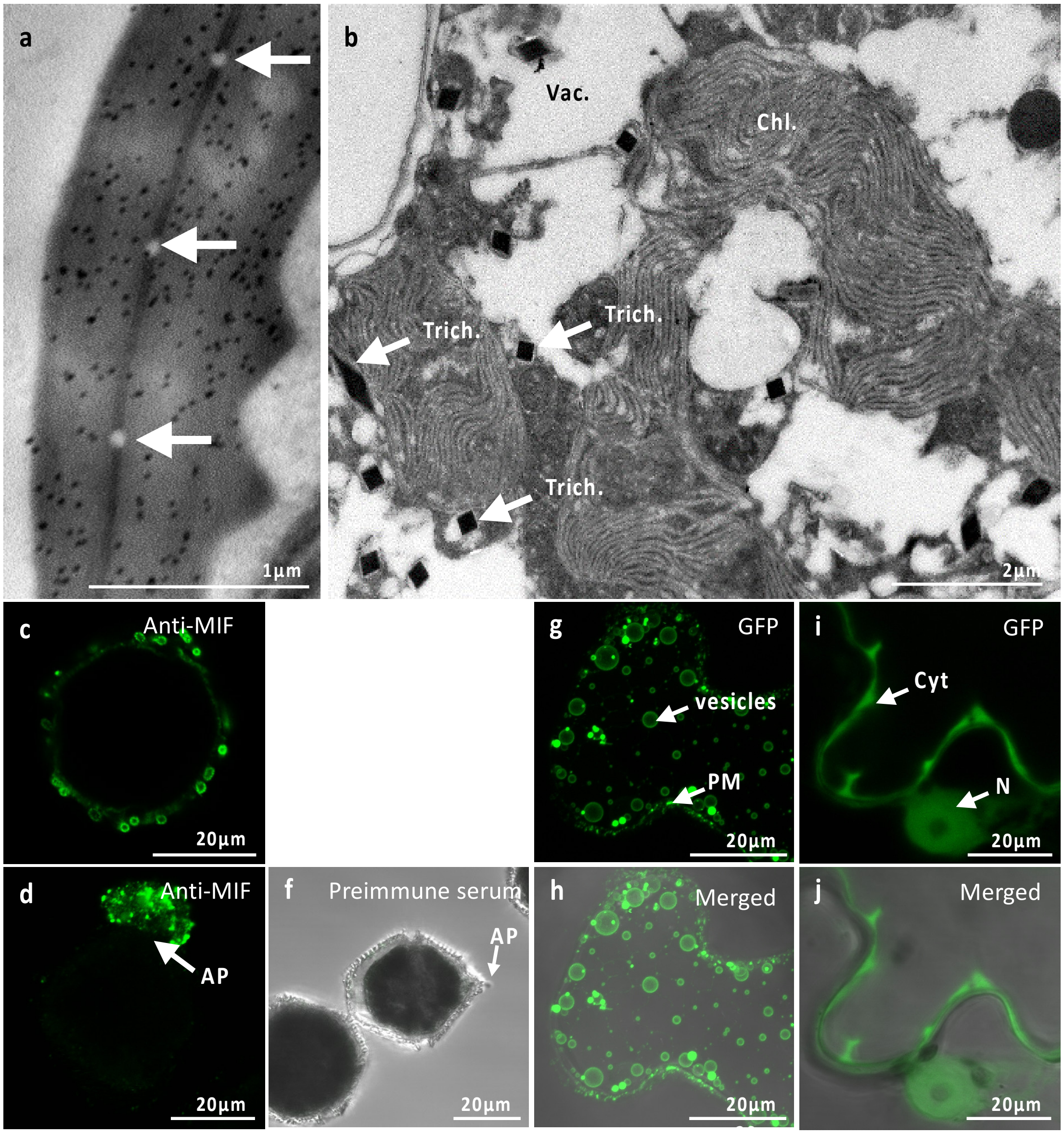

Figure S1. (a,b) Immunogold-labelled electron micrographs of *L. polyedra*. (a) Representative section of the apical pore showing an important accumulation of gold particles (black dots). The secretory pores are indicated by white arrows. (**b)** Representative image of *L. polyedra* cell content showing a vacuole (vac.), chloroplast (chl.) and the

typical sections of the trichocysts (Trich.). Note that no labelled LpMIF (black dots) are observed. **(c,d)** Images corresponding to Figure 2g and h but only with LpMIF labelled in green (Alexa 488®). **(e,f)** Negative control samples directly exposed to the secondary antibody (but no primary antibody) or exposed to pre-immune serum prior to secondary antibody. No labelling was detected in either case. The white arrow indicates the apical pore**. (g-j)** Subcellular localization of LpMIF fused at the N-terminus to GFP, and expressed with (**g,h**) or without the transmembrane domain (**i,j**) in *Nicotiana benthamiana*. (**g,h)** GFP:LpMIF with its endogenous transmembrane domain localizes to vesicle membranes and accumulates in the plasma membrane (PM). **(i,j)** The MIF domain of LpMIF (GFP:LpMIF_TM) without the transmembrane domain localizes to the cytoplasm (Cyt) and the nucleus (N).

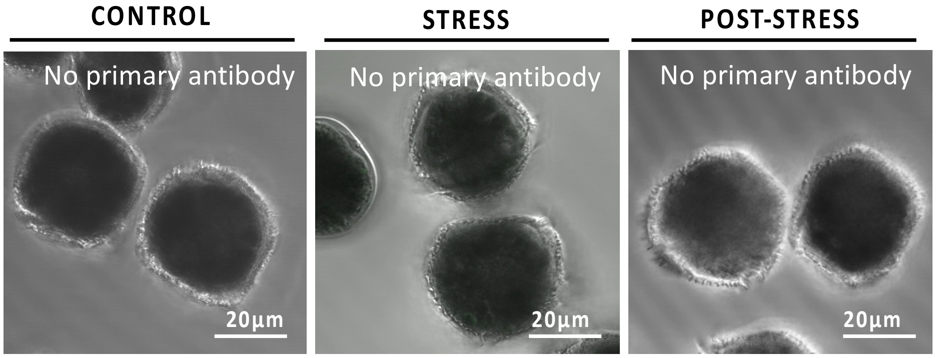

Figure S2. Negative control samples of unstressed (Control) and stressed *L. polyedra* (in the presence of copepods), and after recovery (Post-Stress). Samples were directly exposed to the secondary antibody without the primary antibody.
